# Cardiovascular outcomes with diltiazem vs verapamil in permanent atrial fibrillation

**DOI:** 10.1016/j.hroo.2026.01.027

**Published:** 2026-02-02

**Authors:** Laurent Fauchier, Lisa Lochon, Dylan Pereira, Thibault Lenormand

**Affiliations:** Service de Cardiologie, Centre Hospitalier Universitaire Trousseau et Faculté de Médecine, Université François Rabelais, Tours, France

**Keywords:** Atrial fibrillation, Calcium channel blockers, Diltiazem, Verapamil, Rate control, Heart failure

**Key Findings**

▪ In a large real-world cohort of patients with permanent atrial fibrillation, diltiazem and verapamil were associated with distinct long-term clinical outcomes despite similar guideline indications.
▪ After extensive propensity-score matching, diltiazem was associated with fewer heart failure and cardiovascular hospitalizations compared with verapamil.
▪ Diltiazem showed less durable rate control, with higher rates of tachycardia episodes (heart rate >110 bpm), cardioversion, and atrioventricular node ablation.
▪ Stroke and thromboembolism were similar between treatment groups.
▪ These findings suggest a trade-off between hemodynamic tolerance with diltiazem and durability of ventricular rate control with verapamil, relevant to electrophysiology practice.

Rate control remains a cornerstone of management in patients with permanent atrial fibrillation (AF), yet optimal pharmacologic strategies remain debated.^1^ Non-dihydropyridine calcium channel blockers (NDHP-CCBs), such as diltiazem and verapamil, are commonly used alternatives to beta-blockers, particularly in patients without significant left ventricular systolic dysfunction.^2^, ^3^, ^4^ Although both agents are recommended for rate control, their differing electrophysiological effects may translate into different clinical outcomes. Despite widespread use, cardiovascular outcomes have not been directly compared between diltiazem and verapamil. We therefore assessed associated cardiovascular risks among patients with permanent AF in contemporary practice.

Using deidentified electronic health records from the TriNetX Global Network (158 organizations; >150 million patients), we identified adults with permanent AF (International Classification of Disease, Tenth Revision-Clinical Modification I48.2) between 2010 and 2025 newly treated with diltiazem or verapamil, excluding those with systolic heart failure (HF, I50.2). Propensity-score matching (1:1) was performed across demographic, clinical, laboratory, and medication variables (Table 1). Outcomes included all-cause death, ischemic stroke, hospitalization for HF, hospitalization or visit for AF, cardiovascular-related hospitalization, episode of heart rate >110 bpm during follow-up, atrioventricular (AV) node ablation, cardioversion procedures (considered as failures of usual rate control) and incident cancer. Outcomes were assessed from day 1 to 5 years and expressed as annualized rates. To reduce confounding by indication, analyses were restricted to new users of diltiazem or verapamil, with the index date defined as first prescription. Risk ratios (RRs) with 95% confidence intervals (CIs) were calculated within the TriNetX platform using its integrated survival analysis module and reported, as the proportional hazards assumption for Cox regression was not satisfied. Post-matching balance was verified using standardized differences, and incident cancer was included as a negative control outcome. This study used de-identified data, deemed Institutional Review Board-exempt by the TriNetX Ethics Committee, and adhered to the Declaration of Helsinki as revised in 2013.

**Table 1:** Baseline characteristics and outcomes of patients with permanent AF treated with diltiazem or verapamil after propensity score matching

			After propensity-score matching	SD (%)
		Diltiazem	Verapamil	
		(n = 7787)	(n = 7787)	
*Demographics*				
**Age (years), mean ± SD**		75.5 ± 10.5	75.4 ± 9.7	0.9
**Men, n (%)**		4656 (59.8%)	4678 (60.1%)	0.6
**White, n (%)**		6451 (82.8%)	6454 (82.9%)	0.1
**Black or African American, n (%)**		500 (6.4%)	520 (6.7%)	1
**Asian, n (%)**		253 (3.2%)	251 (3.2%)	0.1
**Hispanic or Latino, n (%)**		229 (2.9%)	222 (2.9%)	0.5
**Socioeconomic and psychosocial circumstances, n (%)**		185 (2.4%)	202 (2.6%)	1.4
*Risk factors*				
**Hypertension, n (%)**		6301 (80.9%)	6239 (80.1%)	2
**Diabetes mellitus, n (%)**		3007 (38.6%)	2970 (38.1%)	1
**Smoker, n (%)**		2521 (32.4%)	2515 (32.3%)	0.2
**Overweight or obesity, n (%)**		2375 (30.5%)	2378 (30.5%)	0.1
**Dyslipidemia, n (%)**		5584 (71.7%)	5574 (71.6%)	0.3
**Alcohol-related diagnoses, n (%)**		202 (2.6%)	211 (2.7%)	0.7
*Cardiovascular comorbidities*				
**Heart failure, n (%)**		3549 (45.6%)	3510 (45.1%)	1
**Coronary artery disease, n (%)**		4854 (62.3%)	4895 (62.9%)	1.1
**Myocardial infarction, n (%)**		1346 (17.3%)	1310 (16.8%)	1.2
**Ischemic stroke, n (%)**		1194 (15.3%)	1160 (14.9%)	1.2
**Intracranial hemorrhage, n (%)**		233 (3%)	224 (2.9%)	0.7
**Valve disease, n (%)**		1979 (25.4%)	1938 (24.9%)	1.2
*Non-cardiovascular comorbidities*				
**Kidney disease, n (%)**		2920 (37.5%)	2904 (37.3%)	0.4
**COPD, n (%)**		1474 (18.9%)	1463 (18.8%)	0.4
**Sleep apnea syndrome, n (%)**		1998 (25.7%)	1991 (25.6%)	0.2
**Peripheral vascular disease, n (%)**		1084 (13.9%)	1054 (13.5%)	1.1
**Previous neoplasm, n (%)**		2391 (30.7%)	2360 (30.3%)	0.9
**Cognitive impairment, n (%)**		91 (1.2%)	90 (1.2%)	0.1
*Laboratory tests and examinations*				
**Systolic BP (mm Hg), mean ± SD**		125.3 ± 20.9	127.0 ± 21.6	7.8
**Diastolic BP (mm Hg), mean ± SD**		70.9 ± 13.7	70.7 ± 14.9	1.2
**HR ≥70/min, n (%)**		5488 (70.5%)	5471 (70.3%)	0.5
**HR >100/min, n (%)**		2239 (28.8%)	2226 (28.6%)	0.4
**Body mass index (kg/m^2^), mean ± SD**		30.2 ± 7.7	30.3 ± 7.4	2
**Total cholesterol (mg/dL), mean ± SD**		148.7 ± 42.5	148.4 ± 42.0	0.7
**Estimated GFR (MDRD, ml/min), mean ± SD**		66.3 ± 28.8	65.4 ± 26.6	3.3
**BNP, ng/L, mean ± SD**		1091.9 ± 3300.5	1012.2 ± 2915.8	2.6
**NT-proBNP, ng/L, mean ± SD**		4147.0 ± 7598.2	3599.3 ± 6289.7	7.9
**LVEF, mean ± SD**		56.0 ± 12.4	55.7 ± 11.7	2.7
*Medications*				
**Beta-blockers, n (%)**		5713 (73.4%)	5696 (73.1%)	0.5
**ACE inhibitors, n (%)**		2204 (28.3%)	2188 (28.1%)	0.5
**Angiotensin II inhibitors, n (%)**		1853 (23.8%)	1816 (23.3%)	1.1
**ARNI, n (%)**		75 (1%)	65 (0.8%)	1.4
**MRA, n (%)**		862 (11.1%)	859 (11%)	0.1
**Diuretics, n (%)**		4632 (59.5%)	4556 (58.5%)	2
**Digitalis glycosides, n (%)**		951 (12.2%)	923 (11.9%)	1.1
**Amiodarone, n (%)**		893 (11.5%)	841 (10.8%)	2.1
**Lipid-lowering drugs, n (%)**		5188 (66.6%)	5128 (65.9%)	1.6
**Glucose-lowering therapy, n (%)**		2855 (36.7%)	2749 (35.3%)	2.8
**Antiplatelet therapy, n (%)**		4775 (61.3%)	4796 (61.6%)	0.6
**Warfarin, n (%)**		2168 (27.8%)	2097 (26.9%)	2
**Apixaban, n (%)**		2344 (30.1%)	2382 (30.6%)	1.1
**Rivaroxaban, n (%)**		978 (12.6%)	988 (12.7%)	0.4
**Dabigatran, n (%)**		273 (3.5%)	289 (3.7%)	1.1

***Clinical outcomes (FU 2.6 ± 1.9 years, median 2.5, IQR 4.1)***				
	**Number of events (yearly rate, %)**		**Risk ratio (95% CI)**	***P* value**
**Death**	2449 (8.49)	1872 (6.82)	1.308 (1.243–1.377)	<.0001
**Ischemic stroke or thromboembolism**	1195 (4.40)	1228 (4.26)	0.973 (0.904–1.047)	.47
**Hospitalization for HF**	2072 (6.90)	2486 (7.94)	0.833 (0.794–0.875)	<.0001
**Hospitalization or visit for AF**	4143 (11.99)	4294 (12.39)	0.965 (0.937–0.993)	.02
**Cardiovascular-related hospitalization**	3358 (10.94)	3876 (11.98)	0.866 (0.837–0.896)	<.0001
**HR >110 bpm during FU**	2001 (7.01)	1537 (5.41)	1.302 (1.228–1.380)	<.0001
**AV-node ablation**	90 (0.35)	59 (0.25)	1.523 (1.098–2.112)	.01
**Cardioversion**	542 (1.90)	405 (1.40)	1.338 (1.181–1.516)	<.0001
**Incident cancer**	553 (3.17)	596 (3.23)	0.935 (0.837–1.044)	.23

Values are n (%) or mean ± SD.

ACE = angiotensin converting enzyme; AF = atrial fibrillation; AV = atrioventricular; BP = blood pressure; CI = confidence interval; COPD = chronic obstructive pulmonary disease; eGFR = estimated glomerular filtration rate; FU = follow-up; HF = heart failure; HR = heart rate; ICD = implantable cardioverter defibrillator; IQR = interquartile range; LVEF = left ventricular ejection fraction; SD = standard deviation.

Among 59,114 adults with permanent AF initiating NDHP-CCBs, 7787 patients were matched per group after 1:1 propensity-score matching. Mean age was 75 years, 60% were men, and all baseline demographic characteristics, cardiovascular and non-cardiovascular comorbidities, laboratory parameters, and concomitant therapies were well-balanced, with standardized differences <10% (Table 1). Over a median follow-up of 2.5 years (interquartile range 4.1), annualized event rates showed higher all-cause mortality with diltiazem compared with verapamil (8.5% vs. 6.8%; RR 1.31, 95% CI 1.24–1.38), but lower annualized risks of hospitalization for HF (6.9% vs. 7.9%; RR 0.84, 95% CI 0.80–0.89), AF-related hospitalization or visit (RR 0.96, 95% CI 0.93–0.98), and overall cardiovascular hospitalization (RR 0.86, 95% CI 0.84–0.89). Diltiazem was associated with higher annualized rates of heart rate >110 bpm (RR 1.30, 95% CI 1.23–1.38), AV-node ablation (RR 1.52, 95% CI 1.10–2.11), and cardioversion (RR 1.34, 95% CI 1.18–1.52). Annualized risks of ischemic stroke or thromboembolism and incident cancer were similar between groups (Table 1).

In patients with permanent AF, selection between verapamil and diltiazem is central to electrophysiology practice, as it directly influences ventricular rate control, symptom burden, and escalation to cardioversion or AV-node ablation.^2^ Both NDHP-CCBs slow AV nodal conduction and provide rate control, but differ in pharmacokinetics and negative inotropic effects. In this large real-world cohort of patients with permanent AF, diltiazem use was associated with fewer hospitalizations for HF and cardiovascular causes compared with verapamil, suggesting better hemodynamic tolerance, but also higher rates of episodes with rapid ventricular response, cardioversion, and AV-node ablation—findings consistent with less sustained rate control. Analyses followed an intention-to-treat strategy, as dosing information was unavailable, reflecting pragmatic clinical practice rather than protocol-driven titration.

Importantly, prior observational studies have shown that ventricular rates ≥100–110 bpm in AF are associated with increased risks of HF and mortality, independent of rate-control strategy.^2^^,^^3^ In this context, the higher frequency of tachycardia episodes and subsequent cardioversion or AV-node ablation observed with diltiazem suggests less durable mid-term rate control. This may partially offset its favorable hemodynamic profile and contribute to divergent downstream outcomes despite fewer HF hospitalizations.

Prior short-term studies have shown similar symptom improvement and reductions in natriuretic peptides with both verapamil and diltiazem, with greater rate-slowing effects reported for diltiazem.^5^ In contrast, our real-world findings suggest less sustained rate control with diltiazem over the mid-term, reflected by more frequent tachycardia, cardioversion, and AV-node ablation. This discrepancy highlights possible differences between short-term physiological responses and longer-term clinical outcomes. Our results suggest that choice of NDHP-CCB may influence downstream clinical trajectories beyond acute rate control. Nevertheless, residual confounding related to heart rate burden over time or treatment escalation cannot be excluded despite extensive matching. The higher all-cause mortality with diltiazem was not explained by baseline characteristics and could also reflect unmeasured confounding, treatment selection bias, or competing non-cardiovascular events.

Overall, these findings reveal clinically meaningful distinctions between NDHP-CCBs in AF management and support further investigation into the optimal rate-control agent, dose intensity, and patient selection to improve long-term cardiovascular outcomes in AF.

## Disclosures

LF: consultant or speaker fees of small amounts for AstraZeneca, Bayer, BMS/Pfizer, Boehringer Ingelheim, Boston Scientific, Medtronic, Novo Nordisk, and Zoll outside of this work. The other authors have no conflicts of interest to disclose.
